# Self-Perception Profile, Body Image Perception and Satisfaction in Relation to Body Mass Index: An Investigation in a Sample of Adolescents from the Campania Region, Italy

**DOI:** 10.3390/children11070805

**Published:** 2024-06-30

**Authors:** Giada Ballarin, Francesca Gallè, Lucia Dinacci, Federica Liberti, Antonia Cunti, Giuliana Valerio

**Affiliations:** Department of Medical, Movement and Wellbeing Sciences, University of Naples “Parthenope”, 80133 Naples, Italy; giada.ballarin001@studenti.uniparthenope.it (G.B.); lucia.dinacci001@studenti.uniparthenope.it (L.D.); federica.liberti001@studenti.uniparthenope.it (F.L.); antonia.cunti@uniparthenope.it (A.C.); giuliana.valerio@uniparthenope.it (G.V.)

**Keywords:** body image perception, silhouette test, perceived physical fitness, self-perception profile, body mass index

## Abstract

(1) Background: Body image dissatisfaction has been commonly reported in adolescents with overweight/obesity and has been related to a lack of motivation to change lifestyle behaviors. Plus, a better perception of physical fitness has been related to a higher self-perception in physical aspect and social items in youths. (2) Methods: A total of 120 (59.2% males, mean age of 12.3 ± 0.9 years) middle-school adolescents participated in the present study. Anthropometric measurements were assessed following the standard procedures. Body weight perception and body image satisfaction were assessed using the Silhouette Figure Body Images Test. Self-perception profile, perceived physical fitness, and perceived difficulties in conducting physical tasks were assessed by using structured questionnaires. (3) Results: Overweight was present in 27.5% adolescents and obesity in 32.5%. A total of 89.7% of adolescents with obesity did not have a real perception of his/her weight status and 84.6% of adolescents with obesity were not satisfied with his/her body image. The self-perception of physical appearance, perceived difficulties in running, perceived overall fitness, and perceived speed/agility were lower in adolescents who were unsatisfied with their body image. Body image satisfaction was positively associated with the self-perception of physical appearance and overall fitness and negatively associated with perceived difficulties in running, independently of sex, age, and BMI. (4) Conclusions: Adolescents with obesity did not have a real perception of their weight status and were unsatisfied with their body image. The positive association between body satisfaction, a better perception of overall fitness, and less difficulties in conducting physical tasks suggests the protective role of the components of physical activity and health-related fitness on body acceptance in a critical period of life.

## 1. Introduction

Adolescence has been recognized as a crucial period for the development of body image [1]. Furthermore, several studies have also shown the role of body image satisfaction in determining and maintaining the concept of self during this period [2,3,4]. During adolescence, self-perceived body image can be influenced by family and cultural stereotypes, peer interactions, media, and social models [5]. When deviations from the expectations related to social models occur, the concept of self can be undermined [3]. An inaccurate perception of individuals’ own body size, named body size misperception, is frequent in adolescents with overweight and obesity and can negatively affect motivation to change health-related behaviors, such as diet and physical activity (PA) [6,7]. Insufficient PA, which contributes to overweight and obesity, is in fact associated with body image perception and satisfaction among adolescents: recent findings show that, as PA levels increase, dissatisfaction decreases [8]. Furthermore, the ability to adopt and maintain healthy behaviors, including regular PA, may be compromised by poor psychosocial health, in particular by a lower self-efficacy [9]. Objective or perceived motor competence is closely related with PA levels and physical fitness [10,11]. In fact, increasing PA levels and fitness in adolescents with overweight and obesity has been proven to be effective in improving their perceived physical competence [12].

In addition, evidence shows that obesity can coexist with eating disorders in a strict relationship with body satisfaction [13,14]. Unhealthy eating behaviors, such as binge eating, are frequent in youths with overweight and obesity and can lead to the onset of the clinical symptoms of eating disorders. Body dissatisfaction has been identified as a cognitive risk factor for both obesity and eating disorders, with weight-related stigma, mainly weight-related teasing by parents and peers, acting as a mediator between obesity and eating disorders. In fact, bulimia nervosa and binge eating disorder may be a result of failed attempts to control weight in adolescents with obesity and impaired psychosocial wellbeing [15]. Due to these relationships, the possible presence of body dissatisfaction and the risk of eating disorders should be considered in obesity management to avoid treatment failure and related complications [14,16]. Integrated interventions based on both diet and physical activity are currently recommended as the best strategy to prevent or treat obesity in childhood and adolescence [17]. However, it should be considered that an energy balance approach can increase the risk of developing eating disorders and cannot be effective in maintaining weight status in the long term [18]. The WHO Commission on Ending Childhood Obesity report suggests that these interventions should include psychosocial and family support in addition to common components such as nutrition and PA or sedentary behavior change [19]. Evidence shows that energy-balance interventions, including physical activity, mainly resistance exercise, in addition to diet changes, are effective in improving anthropometric parameters and body composition by increasing lean mass. However, these effects are frequently observed at post-intervention, but not in the long term [18,20], suggesting that other risk factors for both obesity and eating disorders, such as body image and satisfaction, and eating behaviors and teasing, should be also evaluated [21,22]. Therefore, investigating the relationship between weight status, body image and satisfaction, and perceived competence is fundamental to provide policy makers with information regarding the psychosocial elements that need to be addressed in the development of interventions for obesity prevention and management. This study aimed to assess self-perceived body image and body satisfaction in a sample of adolescents from southern Italy with reference to their objectively measured weight status. Furthermore, we aimed to evaluate the possible association between body image perception and satisfaction, perceived difficulties in physical tasks, perceived physical fitness, and self-perception profile.

## 2. Materials and Methods

Two middle schools of Naples, Italy, joined this study, with a cross-sectional design. Parents or legal guardians and adolescents were asked to sign a written informed consent for procedures before joining the study. The Ethical Committee of the University of Campania “Luigi Vanvitelli” (protocol number n. 8383/2023) approved the present study. Among the 172 students attending the selected school (57% boys and 43% girls), 44 did not provide consent to join the study, while 8 were absent on the day of the measurements. Summarizing, 120 (59% boys and 41% girls) joined the present study (69.8% of the eligible sample). 

### 2.1. Anthropometric Measurements

Anthropometric measurements were performed by the same skilled operator according to standard procedures. The participants worn light clothes, without shoes. Body weight was measured to the nearest 0.1 kg using a medical portable scale (SECA 813) and height was measured to the nearest 0.5 cm using a portable stadiometer (SECA 217). BMI was defined as weight/stature^2^ (kg/m^2^) and transformed into BMI Z-scores according to the WHO growth reference [23]. A BMI Z-score < −2.0 corresponds to underweight, BMI Z-score between −2 and +0.99 to normal weight, BMI Z-score ≥ 1 to overweight, and BMI Z-score ≥ 2 to obesity.

### 2.2. Questionnaires

Questionnaires were administered through interview by a trained researcher in a separate room. They included the following items.

1. Weight status perception: The “Silhouette Figure Body Images Test” [24] was used to assess the correspondence between perceived and real weight status and validated in Italian adolescents by Lombardo et al. [25]. The images were classified as follow: A and B for underweight; C and D for normal weight; E and F for overweight; and G and H for obesity. The participants were asked to identify their image among those provided. Comparing the response with the real weight status, the participants were classified as RIGHT when the chosen image corresponded to the real weight status and WRONG when the image did not correspond to the real weight status.
2. Body satisfaction: The “Silhouette Figure Body Images Test” [24,25] was used to assess body satisfaction. Each participant selected the figure she/he thought corresponded to their real image (a) and then selected the figure they thought corresponded to their ideal image (b). The difference between a and b was calculated and the participants were then divided into 2 groups: satisfied (B-SAT) when the difference was 0 and unsatisfied (B-DIS) when the difference was lower/higher than 0.
3. Perceived physical fitness: The International Fitness Scale (IFIS), created and validated by Ortega et al. [26] to assess the individual perception of physical fitness, is composed of five questions according to the following macro-areas: general fitness, cardiorespiratory fitness, strength, speed–agility, and flexibility. Answers were provided along a five-point Likert scale, where 1 corresponded to “very poor” and 5 to “very good”.
4. Self-perception profile regarding social competence, motor competence, and physical appearance was assessed by a tool designed and validated by Harter and validated in an Italian sample by Aleni Sestito et al. [27,28]. Each domain consisted of six questions, to which 1 to 4 points were ascribed, with a total minimum score of 6 and a maximum score of 24 points.
5. Perceived difficulties with physical tasks were assessed through a structured questionnaire, exploring physical difficulties related to the following daily tasks: walking, running, and jumping [29]. Four possible answers (0 = never; 1 = sometimes; 2 = often; 3 = always) were available for each item. Perceived difficulty was defined whether the perception was ≥1 in each item [29,30].
6. Physical activity and sedentary time: The participants were asked to report about sports engagement in the previous 6 months (yes/no), the type of sport, the weekly hours spent in organized (sports) and/or non-organized (walking to school, walking the dog, playground activities, cycling, skateboarding, roller blading, or dancing) physical activities, and the daily hours spent in sedentary time (sitting or lying down). During the interview, examples were provided by the researchers.

### 2.3. Statistical Analysis

Data are expressed as means and standard deviations or medians and interquartile ranges or frequencies (%) and 95% confidence interval (CI). All variables had skewed distribution. Between-groups differences were evaluated by using the Mann–Whitney test. Chi-squared or Fisher’s exact tests, as appropriate, were used to compare proportions. Adjusted binary logistic regression analyses were used with or without corresponding 95% confidence intervals, which were calculated to evaluate the association of weight perception or body image satisfaction and the variables of interest (such as perceived physical fitness, perceived difficulties in physical tasks, or lifestyle variables). A *p*-value < 0.05 was considered statistically significant. The statistical analysis was performed using IBM SPSS Statistics, Version 28.0 (Armonk, NY, USA: IBM Corp.). 

## 3. Results

The general characteristics of the sample stratified by weight perception or body image satisfaction are shown in Table 1. The BMI Z-score was significantly higher in adolescents with wrong weight perception, while no statistical differences were found for sex, age, and BMI Z-score when body satisfaction groups were considered. The frequency comparison among the BMI categories showed that 89.7% of adolescents with obesity had a wrong weight perception. Similarly, the 84.6% of adolescents with obesity were unsatisfied with their own body image (*p* = 0.014). 

Self-perception profile, perceived difficulties in conducting physical tasks, perceived physical fitness, and lifestyle behaviors stratified for weight perception categories are shown in Table 2. The self-perception of physical appearance, perceived flexibility, and hours of physical activity were lower in adolescents with a wrong weight perception. The same variables were compared between groups of body image satisfaction (Table 3). The self-perception of physical appearance, perceived difficulties in running, perceived overall fitness, or perceived speed/agility were lower in adolescents who were unsatisfied with their body image, while no statistical significance was present for the other considered variables.

Different models of logistic regression analyses were run in order to analyze the possible predictors of perceived weight or body image satisfaction, adjusting for sex, age, and BMI Z-score.

A wrong weight status perception was positively associated with the BMI Z-score and negatively associated with the perception of flexibility and hours of physical activity, independently of sex, age, and BMI (Table 4). Body image satisfaction was positively associated with the self-perception of physical appearance and overall fitness and negatively associated with perceived difficulties in running (Table 5).

## 4. Discussion

The prevalence of overweight and obesity in children and adolescents has alarmingly increased worldwide [31], especially after the COVID-19 pandemic [32,33]. In Italy, a mean prevalence of overweight/obesity equal to 22.6% was reported in 2022 for the adolescent population, with geographical differences. Higher prevalence rates were reported for Southern regions, and among these, the Campania region accounted for the highest (31.6%) [34]. Obesity in adolescents is worthy of attention since its early development increases the risk of developing physical and psychological disorders that may persist later in life [31]. Healthy diet and active behaviors are fundamental instruments for prevention strategies, but their implementation requires also awareness on weight perception, body image satisfaction, and motor competence. In our study, wrong weight perception and body image dissatisfaction were found to be more common among adolescents with overweight and obesity (Table 1). In particular, compared to previous studies, we found a higher percentage of unsatisfied adolescents with overweight/obesity (78% vs. 66% or 61%) [35,36]. Furthermore, a wrong weight status perception was associated only with lower perceived flexibility and PA, while body image dissatisfaction was related with lower self-perceived physical appearance and overall fitness.

The literature shows that weight underestimation is common not only among children and adolescents with overweight/obesity, but also among their caregivers, and it is influenced by parental weight status, ethnicity, and educational level [37,38]. Although parents were not investigated in this study, data obtained in the same districts where the two schools are located, within the surveillance system of Italian children’s health “OKkio alla Salute”, highlight that the 75% of parents with children with overweight/obesity perceived their child’s weight status as normal [39]. Therefore, it is feasible that the wrong weight perception of children in our sample may be explained by the influence of parental perception and the cultural insights involving southern Italy populations. At the same time, our results are in line with previous evidence reporting a correlation between obesity and body dissatisfaction [40], even though some investigations performed in Mediterranean urban teenagers were not able to find any association between body satisfaction and weight status [41]. However, even people with overweight who correctly perceived their weight status were more likely to develop a poorer psychological wellbeing and worse weight-related outcomes overtime, as reported by Robinson et al. in their systematic review [42]. The explanatory model proposed suggests that even overweight self-perception leads to social rejection concerns and the internalization of weight stigma, which in turn may generate psychological distress and affect the adoption of healthy behaviors that can, in part, explain the progression from overweight to obesity. Therefore, an accurate identification of one’s own weight status does not always account for better health outcomes. Public health interventions should comprehend more weight-inclusive approaches removing stigmatizing language or weight outcomes, in order to obtain a better adherence to health-protective behaviors, such as PA, for all the individuals regardless of their weight status. As matter of fact, previous studies showed an inverse relationship between low PA and body image perception [8,43], but these data were not confirmed in our sample, while the association with perceived physical appearance deserves further research. Interestingly, intervention studies based on exercise were able to improve the negative body image in different age groups [44].

Our findings highlight that body image was related with flexibility and body satisfaction was found to be related with perceived physical appearance and overall fitness. The association between body satisfaction and perceived fitness in adolescents has been previously reported [45,46]. Therefore, our data support that the increase in physical fitness and improvement in the quality of life, more than weight loss, might be considered as index of good compliance [47]. Furthermore, an inverse relationship between PA and body weight perception was found. A reciprocal relationship between body image and PA or exercise has been shown in adolescents, in whom body image was a predictor of moderate–vigorous PA regardless of BMI, and exercise-based interventions have the potential of improving body image [48,49]. Furthermore, some evidence shows that PA can influence the relationship between BMI and body image [50]. 

The limitations of this study lie in the design of the study and in the characteristics of the sample. Indeed, given its cross-sectional nature, the study does not allow for the establishment of the direction of causal relationships between the variables examined. As for the sample, both its small size and the enrolment performed by convenience limit its representativeness for the adolescent population. However, all the surveys used in this study were validated in Italian adolescents. 

Considering the high prevalence of overweight/obesity shown by this population from southern Italy, our results may be useful to highlight the need of educational interventions addressed to the local population in order to support adolescents in developing a healthy body image and adopting healthy behaviors. More insights are needed to explain the discrepancy found in the association between body dissatisfaction and self-perception profile sub-scales.

## 5. Conclusions

Healthcare professionals should consider body size misperception when they work with adolescents, particularly if they are overweight/obese, and provide a personalized approach to support the change in lifestyle behaviors. Since perceived fitness and motor competence are strictly related to PA levels and body satisfaction, intervention strategies should include not only nutritional support and weight-centered approaches, but they should also aim to improve physical activity and physical fitness in order to trigger a so-called “positive spiral of engagement”.

## Figures and Tables

**Table 1:** General characteristics of the sample grouped by weight perception or body satisfaction.

	Weight Perception			Body Image Satisfaction		
	Right n = 52	Wrong n = 68	*p*-Value	Satisfied (n = 35)	Unsatisfied (n = 85)	*p*-Value
Sex, males n (%)	52 (56)	68 (63)	0.412	23 (66)	48 (56)	0.353
Age (years)	12.4 ± 0.9	12.2 ± 0.9	0.230	12.3 ± 0.9	12.3 ± 0.9	0.902
BMI Z-score	0.67 [−0.2–1.3]	2.0 [1.1–2.5]	**<0.001**	1.0 [0.3–1.8]	1.6 [0.4–2.3]	0.081
**BMI Categories**						
Normal weight (n = 48)	34 (70.8)	14 (29.2)	**<0.001**	20 (41.0)	28 (59.0)	**0.014**
Overweight (n = 33)	14 (42.4)	19 (57.6)		10 (30.3)	23 (69.7)	
Obese (n = 39)	4 (10.3)	35 (89.7)		6 (15.4)	33 (84.6)	

**Table 2:** Self-perception profile, perceived physical fitness, and perceived difficulties in conducting physical tasks in weight perception categories.

	RIGHT Body Perception (n = 52)	WRONG Body Perception (n = 68)	*p*
**Self-perception profile**			
Social competence	3.2 [2.5–3.7]	3.0 [2.5–3.5]	0.284
Physical appearance	3.0 [2.2–3.5]	2.5 [1.7–3.3]	**0.038**
Motor competence	2.7 [2.2–3.0]	2.3 [2.0–3.1]	0.284
**Perceived difficulties in conducting physical tasks**			
Walking	1.0 [1.0–1.0]	1.0 [1.0–1.8]	0.354
Running	1.0 [1.0–2.0]	1.0 [1.0–3.0]	0.249
Jumping	1.0 [1.0–1.0]	1.0 [1.0–1.0]	0.592
**Perceived physical fitness**			
Overall fitness	3.0 [3.0–4.0]	3.0 [2.0–4.0]	0.092
Musculoskeletal strength	3.0 [2.0–4.0]	3.0 [2.3–4.0]	0.621
Cardiorespiratory fitness	4.0 [3.0–4.0]	4.0 [3.0–4.0]	0.264
Speed/agility	4.0 [3.0–5.0]	4.0 [3.0–4.0]	0.211
Flexibility	4.0 [3.0–4.0]	3.0 [2.0–4.0]	**0.024**
**Lifestyle behaviors**			
Sport practice n (%)	37 (71.2)	33 (48.5)	0.010 *
Weekly hours of physical activity	3.0 [1.0–4.0]	2.0 [0.0–3.0]	**0.033**
Daily hours of sedentary habits	3.0 [2.0–4.0]	3.0 [2.0–4.0]	0.154

* Chi-squared.

**Table 3:** Self-perception profile, perceived physical fitness, and perceived difficulties in physical tasks in body satisfaction/dissatisfaction categories.

	Body Image Satisfied (n = 35)	Body Image Dissatisfied (n = 85)	*p*-Value
**Self-perception profile**			
Social competence	3.3 [2.5–3.5]	3.0 [2.5–3.5]	0.502
Physical appearance	3.3 [2.5–3.7]	2.4 [2.5–3.2]	**<0.001**
Motor competence	2.5 [2.2–3.2]	2.5 [2.0–3.0]	0.318
**Perceived difficulties in conducting physical tasks**			
Walking	1.0 [1.0–1.0]	1.0 [1.0–2.0]	0.171
Running	1.0 [1.0–2.0]	1.0 [1.0–3.0]	0.050
Jumping	1.0 [1.0–1.0]	1.0 [1.0–1.0]	0.729
**Perceived physical fitness**			
Overall fitness	4.0 [3.0–4.0]	3.0 [2.0–4.0]	**0.008**
Musculoskeletal strength	4.0 [2.0–4.0]	3.0 [3.0–4.0]	0.868
Cardiorespiratory fitness	4.0 [3.0–5.0]	4.0 [3.0–4.0]	0.076
Speed/agility	4.0 [3.0–5.0]	4.0 [3.0–4.0]	**0.037**
Flexibility	4.0 [3.0–4.0]	3.0 [2.0–4.0]	0.123
**Lifestyle behaviors**			
Sport practice n (%)	19 (54.3)	51 (60.0)	0.353 *
Weekly hours of physical activity	2.0 [0.0–4.0]	2.0 [0.0–4.0]	0.591
Daily hours of sedentary habits	3.0 [2.0–4.0]	3.0 [2.0–4.0]	0.314

* Chi-squared.

**Table 4:** Odds ratios from logistic regression models with wrong weight status perception as the outcome, adjusted for sex, age, and BMI Z-score.

Models	Variables	OR	95% C.I.		*p* Value
1st	BMI Z-score *	2.320	1.462	3.190	**<0.001**
2nd	BMI Z-score *	2.241	1.533	3.275	**<0.001**
3rd	Perceived flexibility *	0.663	0.443	0.991	**0.045**
	BMI Z-score *	2.388	1.600	3.565	**<0.001**
	Hours of physical activity *	0.642	0.474	0.868	**0.004**

* Sex, age, and BMI Z-score were covariates in all models.

**Table 5:** Odds ratios from logistic regression models with body image satisfaction as the outcome, adjusted for sex, age, and BMI Z-score.

Models	Variables	OR	95% C.I.		*p* Value
1st	Physical appearance *	0.380	0.209	0.690	**0.001**
2nd	Overall fitness *	0.531	0.326	0.864	**0.011**
3rd	Perceived speed/agility	0.727	0.458	1.153	NS

* Sex, age, and BMI Z-score were covariates in all models.

## Data Availability

The data presented in this study are available upon request from the corresponding author.
